# Advances in the Chemistry of Porphyrins and Related Macrocycles

**DOI:** 10.3390/ijms22147487

**Published:** 2021-07-13

**Authors:** Andrea Romeo, Maria Angela Castriciano, Luigi Monsù Scolaro

**Affiliations:** 1 Dipartimento di Scienze Chimiche, Biologiche, Farmaceutiche ed Ambientali, University of Messina and C.I.R.C.M.S.B, V.le F. Stagno D’ Alcontres, 31-98166 Messina, Italy; 2 CNR-ISMN Istituto per lo Studio dei Materiali Nanostrutturati c/o Dipartimento di Scienze Chimiche, Biologiche, Farmaceutiche ed Ambientali, University of Messina, V.le F. Stagno D’ Alcontres, 31-98166 Messina, Italy

Porphyrins and their analogues feature remarkably in nature, being prosthetic groups in a wide variety of primary metabolites playing a pivotal role in many biological processes. This class of compounds is especially versatile, being engaged in a wide multitude of today’s core applications ranging from biology to medicine and material science. The Special Issue entitled “Advances in the Chemistry of Porphyrins and Related Macrocycles“ collects featured research papers providing an overview of the recent developments and advances related to some topic aspects of porphyrin and porphyrinoid chemistry, including biological and biomedical applications [1,2,3,4,5,6,7], photophysics [8], supramolecular chemistry [9], and sensing materials [10,11,12]. The Special Issue welcomed the submission of original full research articles [1,3,4,6,7,8,9,10,11], as well as some review articles [2,5,12] aiming to provide a comprehensive foundation on some of the above-mentioned research field, highlighting the current state of knowledge. Several researchers from many research institutions, by virtue of their qualified scientific expertise, have contributed to the success of this Special Issue, and their scientific contributions are summarized here.

Molnar et al. [1] reported on a synthetic procedure finalized to extend the *meso*-phenothiazinyl-phenyl-porphyrin (MPP) dyes family through the preparation of phenothiazine-bridged porphyrin-(hetero)aryl dyads by Suzuki–Miyaura cross-coupling between BrMPP and (hetero)arylboronic acid pinacolates. Three novel synthesized phenothiazine-bridged porphyrin-heteroaryl dyads displaying fluorescence emission above 650 nm were selected to evaluate their biological activity. According to cell viability colorimetric assays (MTT and Alamar Blue), they exhibit a moderate cytotoxicity on ovarian tumor cell lines in vitro (OVCAR-3, cisplatin-sensitive A2780, and cisplatin-resistant A2780cis, respectively). Visualization of the stained living cells was performed both by fluorescence microscopy imaging and by fluorescence lifetime imaging under two-photon excitation (TPE-FLIM), confirming the cellular uptake and the capability of staining the cell nucleus.

Montaseri et al. [2] authored an interesting review article highlighting the last advances on a wide range of porphyrin-based inorganic nanoparticles developed for phototherapy nanotheranostic cancer treatment over the last three years (2017 to 2020).

Authors successfully focused on targeting this article as a current update based on previous reviews, putting a large amount effort in outlining advances and current challenges in the development and future perspectives of porphyrin-based nanomedicines within photodynamic diagnosis (PDD), photothermal therapy (PTT), and photodynamic therapy (PDT) application for cancer treatment.

Within the same framework, Pucelik et al. [3] developed a new class of amphiphilic photosensitizers for photodynamic therapy (PDT). A series of amphiphilic polyfluorinated sulfonate ester porphyrins with four different substitution patterns were characterized in terms of their photochemistry, cellular uptake, subcellular localization, dark cytotoxicity, and photodynamic effect. All the experimental findings prompt the potential use of these novel porphyrin derivatives as new leads for PDT photosensitizers with an appropriate amphiphilicity and remarkable phototoxicity.

Sułek et al. [4] evaluated the physicochemical properties of six differently charged *meso*-tetraphenylporphyrin derivatives and their ability to inactivate the Gram-negative and Gram-positive bacteria in a planktonic suspension under blue light irradiation. It was highlighted as the structural modification of porphyrins influences both photophysical and biological activity in terms of efficacy of photodynamic photoinactivation. In addition, theoretical calculations were performed to explain the distribution of the molecular charges in the evaluated compounds.

Photoacoustic imaging (PAI) is an emerging non-invasive biomedical imaging technique based on optical excitation and ultrasonic detection, having broad application prospects. With this regard, Merkes et al. [5] wrote an attractive review article with the aim of providing an outline of the basic PAI principles as well as to focus the current state of the art in PAI applications using probes based on tetrapyrroles and their related compounds. The authors, highlighting the importance of the application of strong NIR-absorption probes, elaborate on the challenges and future perspectives of current PA probes used for molecular detection and in vivo bioimaging.

Protoporphyrinogen IX oxidase (PPO) is a critical enzyme playing a significant and pivotal role across life, catalyzing the six-electron oxidation of protoporphyrinogen IX (protogen) to protoporphyrin IX (proto) in the last common step of the process, leading to heme biosynthesis.

The lack of structural and mechanistic understanding of substrate recognition represents a longstanding fundamental question in porphyrin biology, and in this respect, Barker et al. [6] created a novel docking model for protogen in the catalytic domain of PPO. This study used a protogen structure derived from the crystal structure of a close precursor in the pathway, coproporphyrinogen III (copro) bound to the catalytic domain of uroporphyrinogen decarboxylase; the intermediates in the pathway were created on the same backbone structure for docking, and the product proto was created on the basis of previous crystallographic data. The docking results support a reaction mechanism proposed previously, whereby all hydride abstractions from C10 of the substrate are followed by tautomeric rearrangements, allowing for the next reaction.

Olson et al. [7] reported on the effects of a wide variety of quinones on sulfur metabolism using H_2_S- and polysulfide-specific fluorophores (AzMC and SSP4, respectively) and thiosulfate-sensitive silver nanoparticles (AgNP). In particular, it has been shown that the ability of hydroxybenzenes to oxidize H_2_S varies greatly with the location and distribution of the hydroxyl groups, and the addition of specific side groups can further change the responsiveness of these compounds. In addition, some biogenic amines such as epinephrine and norepinephrine may reliably oxidize H_2_S, suggesting potential additional roles for these signaling molecules.

Within photophysical studies of biologically relevant chromophores, Fresch and Collini [8] performed a detailed and systematic survey on the relaxation dynamics in the ultrafast timescale of chlorophyll b (chl_b_), a naturally important photosynthetic pigment whose role is pivotal in many functionalities of photosynthetic proteins. Relaxation dynamics have been characterized by 2D electronic spectroscopy (2DES) at room temperature (RT) and at 77 K to undercover the short time dynamics (≈100 fs). The reported studies, whose represent the first report on chl_b_ in this timescale, highlighted significant differences in the internal conversion mechanism between chl_a_ and chl_b_, in agreement with previous reports, therefore suggesting a lower degree of Q_x_–Q_y_ mixing in chl_b_ with respect to chl_a_. At low temperature, when the spectral diffusion due to the inertial component of solvation is hindered, the primary mechanism dominating the first stages of the relaxation of the excited state is an ultrafast redistribution of energy into vibrational modes. This paper offers a valuable insight into an important issue as the characterization of dynamic and mechanistic details of great help to untangle the complex dynamics of chlorophyll molecules in biological light-harvesting complexes.

J-aggregates of 5,10,15,20-tetrakis-(4-sulfonatophenyl)-porphyrin (TPPS_4_) porphyrin are attractive nanomaterials, since by depending on the experimental conditions, protocol of preparation and the eventual use of templating agents, they show a variety of different morphologies and physical-chemical properties. In this framework, Occhiuto et al. [9] reported a detailed kinetic investigation on the formation of TPPS_4_ J-aggregates following the demetallation of the zinc porphyrin derivative as a function of pH and ionic strength. The authors highlighted (i) a proper choice of the experimental conditions, allowing for an accurate control of the free protonated monomer load released over time in solution, and (ii) zinc cation’s role being not only to merely control the ionic strength of the medium, but even to stabilize the nanoaggregates, thus providing the formation of a mesoscopic network responsible for the exhibited peculiar chiroptical properties.

Chemical sensors represent one of the most promising application fields for porphyrin nanostructures, and in this regard, Gaeta et al. [10] reported on the environmental-friendly synthesis of porphyrin functionalized TiO_2_ nanomaterials and their evaluation as an effective tool in the photodegradation of antibiotics. The authors performed a screening of a wide variety of both anionic and cationic porphyrins (respectively, TCPP and TMPyP derivatives) in testing the photodegradation process of two antibiotics, namely, oxolinic acid (OXA) and oxytetracycline (OTC), in aqueous solution at micromolar concentrations. Under simulated solar irradiation, CuTCPP@TiO_2_ appeared to be a promising system for the photo-removal of oxytetracycline from water, by opening the way for these functionalized nanomaterials to constitute an efficient new approach to struggle against antibiotics pollution.

Porphyrin-functionalized silica hybrid materials find remarkable application in many fields such as detection of a large number of substances, as well as removal of toxic heavy metals and catalysis. Here, Anghel et al. [11] describe the synthesis of novel porphyrin-silica hybrid materials in which they embedded Pt(II)-5,10,15,20-tetra-(4-allyloxy-phenyl)-porphyrin (PtTAOPP) and platinum nanoparticles (PtNPs) alone or together with 5,10,15,20-tetra-(4-allyloxy-phenyl)-porphyrin (TAOPP) into tetraethylorthosilicate (TEOS)-based silica matrices. These new multifunctional materials showed high chemical stability, large specific surface areas, and tailored pore shapes so as to be used with an improved performance for the detection/adsorption of carbon dioxide and for the color removal of dyes from wastewater.

Zhen-Li Qi et al. [12] conclude this Special Issue by a comprehensive reviewing on the employment of porphyrins and porphyrin-based materials for metal ion detection. The authors, with the aim of providing the reader a better understanding of the extensive literature on porphyrin composite sensors, proceeded to subdivide the content into five sub-categories as follows: porphyrin film, porphyrin metal complex, metal–organic frameworks, graphene materials, and other materials.

In conclusion, we were very pleased to guest-edit this Special Issue, whose aim has been primarily to collect relevant research papers reflecting the increasingly widespread interest in the chemistry of porphyrins and related macrocycles. We hope this issue could reach the widest audience in the scientific community and it will contribute to boosting further scientific and technological advances into the intriguing world of porphyrins and their related compounds.
